# Reduced IL-17A Secretion Is Associated with High Levels of Pneumococcal Nasopharyngeal Carriage in Fijian Children

**DOI:** 10.1371/journal.pone.0129199

**Published:** 2015-06-12

**Authors:** Edwin Hoe, Laura K. Boelsen, Zheng Quan Toh, Guang Wen Sun, Ghee Chong Koo, Anne Balloch, Rachel Marimla, Eileen M. Dunne, Lisi Tikoduadua, Fiona M. Russell, Catherine Satzke, E. Kim Mulholland, Paul V. Licciardi

**Affiliations:** 1 Pneumococcal Research, Murdoch Childrens Research Institute, Melbourne, Australia; 2 Department of Paediatrics, The University of Melbourne, Melbourne, Australia; 3 School of Applied Science, Republic Polytechnic, Singapore, Singapore; 4 Ministry of Health, Suva, Fiji; 5 Centre for International Child Health, Department of Paediatrics, The University of Melbourne, Melbourne, Australia; 6 The Department of Microbiology and Immunology, The University of Melbourne at the Peter Doherty Institute for Infection and Immunity, Melbourne, Australia; 7 London School of Hygiene and Tropical Medicine, London, United Kingdom; Instituto Butantan, BRAZIL

## Abstract

*Streptococcus pneumonia* (the pneumococcus) is the leading vaccine preventable cause of serious infections in infants under 5 years of age. The major correlate of protection for pneumococcal infections is serotype-specific IgG antibody. More recently, antibody-independent mechanisms of protection have also been identified. Preclinical studies have found that IL-17 secreting CD4+ Th17 cells in reducing pneumococcal colonisation. This study assessed IL-17A levels in children from Fiji with high and low pneumococcal carriage density, as measured by quantitative real-time PCR (qPCR). We studied Th17 responses in 54 children who were designated as high density carriers (N=27, >8.21x10^5^ CFU/ml) or low density carriers (N=27, <1.67x10^5^ CFU/ml). Blood samples were collected, and isolated peripheral blood mononuclear cells (PBMCs) were stimulated for 6 days. Supernatants were harvested for cytokine analysis by multiplex bead array and/or ELISA. Th17 cytokines assayed included IL-17A, IL-21, IL-22 as well as TNF-α, IL-10, TGF-β, IL-6, IL-23 and IFNγ. Cytokine levels were significantly lower in children with high density pneumococcal carriage compared with children with low density carriage for IL-17A (p=0.002) and IL-23 (p=0.04). There was a trend towards significance for IL-22 (p=0.057) while no difference was observed for the other cytokines. These data provide further support for the role of Th17-mediated protection in humans and suggest that these cytokines may be important in the defence against pneumococcal carriage.

## Introduction

The bacterium *Streptococcus pneumoniae* is the most common bacterial respiratory pathogen and causes significant morbidity and mortality in children under the age of five worldwide [1]. An estimated 700,000–1 million deaths per year are attributable to the pneumococcus, most of which occur in developing countries [2]. The primary virulence factor of *Streptococcus pneumoniae* is the polysaccharide capsule, which is the basis of current pneumococcal vaccines [3]. There are 94 pneumococcal serotypes, which express structurally and antigenically different capsular polysaccharides [4]. Pneumococci colonise the nasopharynx and this colonisation can lead to the development of diseases such as meningitis, bacteraemia, sepsis and pneumonia [5].

Protection against pneumococcal infections by current pneumococcal conjugate vaccines (PCVs) is dependent on the production of serotype-specific IgG antibody, which is the major correlate of protection. Although PCVs are highly effective against invasive pneumococcal disease (IPD), they appear to be less effective against mucosal disease and carriage. While protection against IPD is mediated by serotype-specific IgG, the mechanisms of protection against colonisation and mucosal disease are less understood. Recent studies have found that there are factors other than antibodies (capsular and non-capsular) may play a role in protection against pneumococcal colonisation and disease.

Protection against pneumococcal colonisation is mediated by the IL-17A producing Th17 cells [6]. IL-17A signals the recruitment and activation of neutrophils and macrophages to the nasopharynx, leading to the clearance of pneumococci [7]. Many studies have reported a role for CD4+ Th17 cells in reducing pneumococcal colonisation in mouse models. Naive mice lacking CD4+ Th17 cells were not protected against pneumococcal carriage, however immunity was restored with the subsequent transfer of IL-17 secreting CD4+ T cells [8]. In this study, Lu *et al*. further demonstrated the critical role of IL-17A, as IL-17A receptor deficient mice were not protected against pneumococcal disease in contrast to mice lacking in IFNγ or IL-4, which were protected. In another study, the protective effects of Th17 cells were reinforced in which mice depleted of either IL-17A or CD4+ T cells were not able to recruit monocytes and macrophages which are crucial to the clearance of pneumococcus from the mucosal surface [7]. While the role of CD4+ Th17 cells in pneumococcal diseases in animal studies is well supported, it is unclear in humans [7,8,9].

Th17 responses are regulated by regulatory T cells (Tregs) [10]. The importance of Tregs in pneumococcal carriage was unknown until recently. One study found a significant number of Tregs were found in adenoids from children who were positive for pneumococcus compared to those who were negative [11]. This supports the association of pneumococcal carriage in the nasopharynx with increased Treg activity. Other *ex vivo* human studies demonstrated that Tregs possessed an inhibitory effect on pneumococcal specific CD4+ T cell proliferation which further supports their immune-inhibitory role [12]. It is well recognised that the developmental pathways of both Th17 and Treg cell subsets are reciprocally interconnected, and the Th17/Treg balance plays a major role in the regulation of health and diseases [13]. During pneumococcal infections, this balance is disturbed, promoting inflammation. Therefore, this may result in an inverse association between Treg and Th17 responses *in vivo*.

The relationship between IL-17A levels, carriage density and the development of pneumococcal disease is less understood. Given that IL-17A protects against carriage, we were interested in determining whether there was an association between IL-17A levels and carriage density as carriage is a critical precursor for disease development.

As the density of carriage differs widely between individuals, we hypothesized that children with stronger Th17 responses may have lower pneumococcal carriage density. In this study, we examined the Th17 (IL-17A, IL-21, IL-22), Treg (IL-10, TGF-β) and other pro-inflammatory (TNF-α, IL-6, IFN-γ) cytokine profile secreted by peripheral blood mononuclear cells (PBMCs) from Fijian children with high and low densities of nasopharyngeal pneumococcal carriage. We found that elevated levels of Th17 secreted cytokines are associated with children with low pneumococcal carriage, supporting and extending previous murine studies demonstrating the protective roles of IL-17A and Th17-related cytokines against pneumococcal colonisation.

## Material and Methods

### Study population and design

Samples used in this study were from healthy children (N = 65) aged 5–7 years old who were previously vaccinated with various pneumococcal vaccine schedules as part of the Fiji Pneumococcal Project (FiPP) [14] and who had detectable nasopharyngeal carriage density measured by qPCR. We also randomly selected 29 children from this study who did not carry pneumococcus as our control group. The demographics of these children are shown in S1 Table. A follow up study was undertaken to assess long term immune protection in these children. Written informed consent was obtained from the parents/guardians of study participants. Blood and nasopharyngeal swabs were collected from each child. The study was approved by the Fiji National Research Ethics Review Committee and the Royal Children’s Hospital Human Research Ethics Committee. The study was conducted and monitored according to Good Clinical Practice.

### Measurement of carriage

Nasopharyngeal swabs were collected, transported, stored, and processed in line with the World Health Organization guidelines [15,16]. Swabs were placed in 1 mL of Skim Milk tryptone-glycerol-glucose (STGG) medium and stored at -80°C until use. Genomic DNAwas extracted from a 100 μl aliquot of sample using the QIAmp DNA minikit (Qiagen) as previously described [17]. *S*. *pneumoniae* density was examined using quantitative real-time PCR (qPCR) targeting the *lytA* gene using previously published primers and probes [18].

### Blood collection and peripheral blood mononuclear cells (PBMCs) culture

Peripheral blood was collected from participants and PBMCs isolated by Ficoll density gradient centrifugation. *Ex vivo* PBMCs were re-suspended in RPMI-FCS at a density of 2x10^6^ cells/mL and 100 μL/ well seeded in 96 well round bottom plate, then stimulated with an antigenic cocktail containing pokeweed mitogen (83ng/mL), CpG (1.7μg/mL) and *S*. *aureus* Cowan strain for 6 days at 37°C with 5% CO_2_ and 95% humidity. After incubation, supernatants were collected and frozen at -80°C until being assayed for cytokines.

### Measurement of cytokines

Levels of IL-17A, TNF-α, IFN-γ, IL-10 and IL-6 in PBMC supernatants were measured using a multiplex bead array method (Biorad, Hercules, USA). Commercial ELISA kits were used to detect IL-21, IL-22, IL-23 (ebioscience, San Diego, USA) and TGF-β (R&D systems, Minneapolis, USA).

### Statistics

Comparison of carriage density between high and low carriage groups was performed using the Mann-Whitney U test. Cytokine levels between the two carriage groups were compared using the Student’s t test. Cytokines concentrations were log (base e) transformed due to non-normal distribution of the data. The Spearman test was used to determine the correlation between the Th17 cytokines and carriage density. A p<0.05 was considered statistically significant in all cases. Analyses were performed using GraphPad Prism version 5 software package.

## Results

### Relationship between pneumococcal carriage density and IL-17A cytokine levels

**Fig 1:**
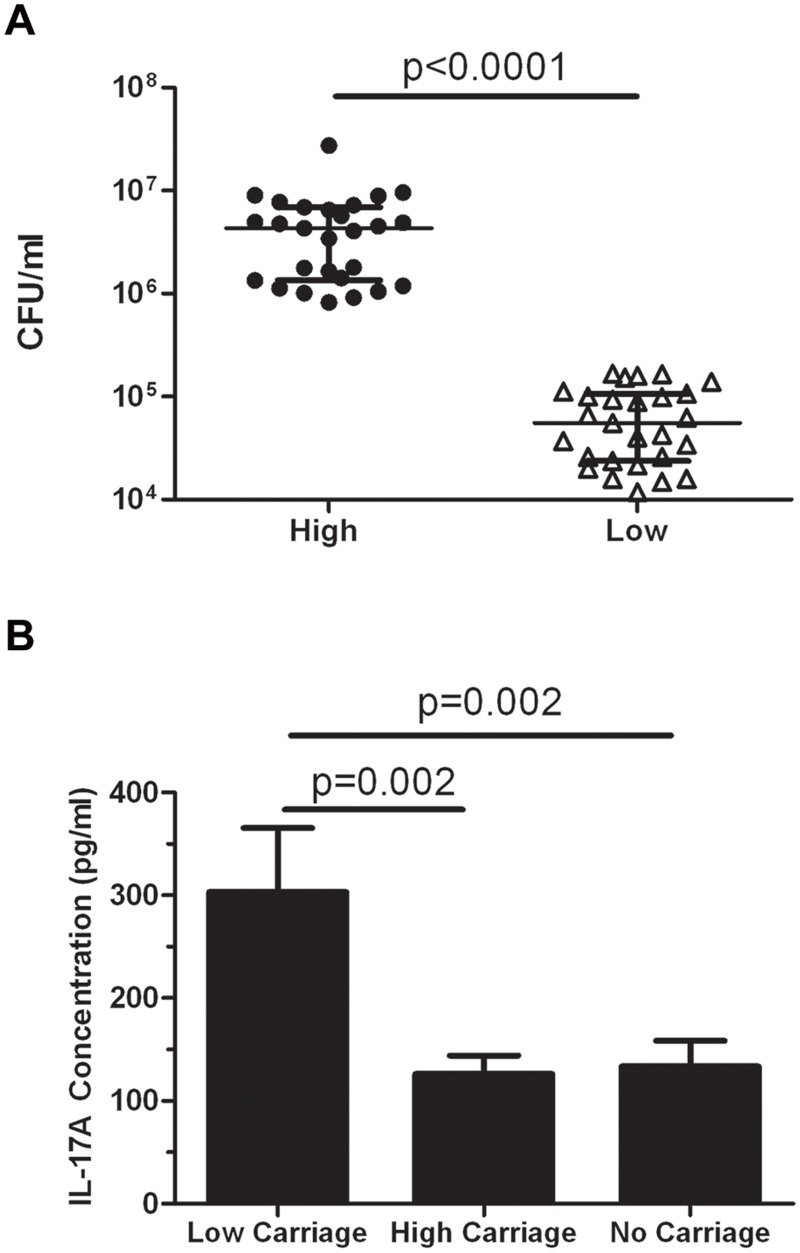
IL-17A levels in children with high and low pneumococcal carriage densities. (A) Carriage density >8.21 x 10^5^ CFU/ml was defined as a ‘high’ carrier and <1.67 x 10^5^ CFU/ml was defined as a ‘low’ carrier. Scatter plots show the median **±** interquartile range for children with high carriage (N = 27) and low carriage (N = 27). Statistical comparisons were done using Mann-Whitney U test. (B) IL-17A levels in PBMC supernatants from children in Fiji with high (N = 27) or low (N = 27) pneumococcal carriage densities as well as children that did not carry pneumococcus (N = 29). Bars represent mean ± SEM. Statistical comparisons were done using an unpaired Student’s t test.

To determine the role of IL-17A, we separated children (N = 65) into those with the highest (N = 27) and lowest (N = 27) pneumococcal carriage densities, resulted in defining our thresholds as >8.21x10^5^ CFU/ml (‘high’ carriage density) and <1.67x10^5^ CFU/ml (‘low’ carriage density). Children with mid-level colonisation were excluded for this analysis (N = 11). There was a significant difference in carriage density between these two groups (p<0.0001; Fig 1A). There was a significant 2.4-fold higher IL-17A level in the low carriage density group (303.3±62.1 pg/ml) compared with the high carriage density group (126±18.2 pg/ml; p = 0.002; Fig 1B). There were also significantly higher IL-17A levels in the low carriage group in comparison with children who did not carry pneumococci (p = 0.002; Fig 1B). No difference in IL-17A levels were observed between children in the high and no carriage groups (Fig 1B).

**Fig 2:**
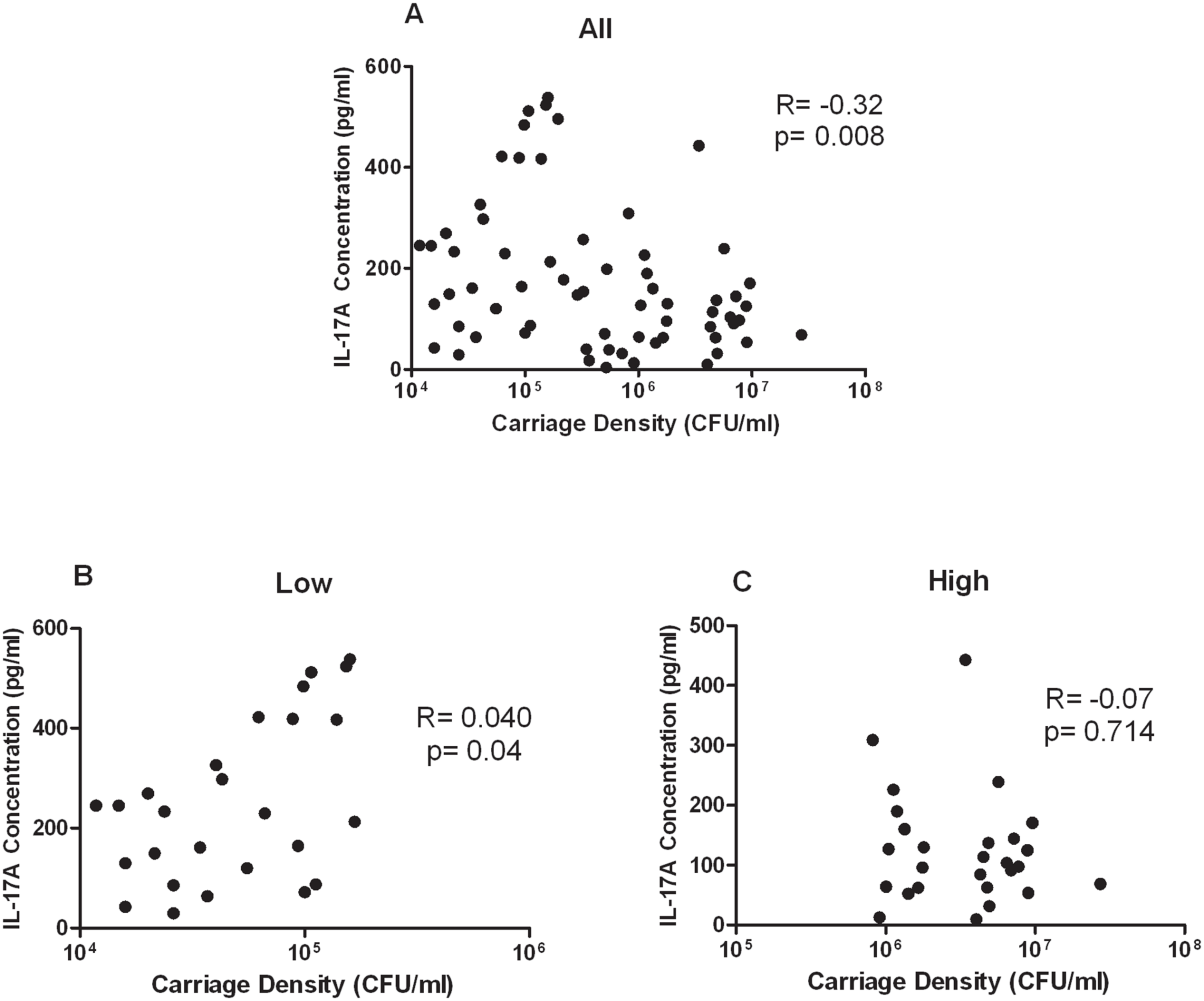
Relationship between pneumococcal carriage densities and IL-17A levels (A) Pneumococcal nasopharyngeal (NP) carriage densities in all 65 children from Fiji was measured by qPCR. IL-17A was measured by multiplex bead array. One child was removed from the analysis as an outlier (Carriage density = 1.65x10^5^ CFU/ml, IL-17A = 1710pg/ml). The correlation with the outlier was R = -0.19, p = 0.126. Correlation between IL-17A and children with low pneumococcal carriage density (B; N = 27) or children with high pneumococcal carriage density (C; N = 27). The Spearman test was used to correlate children with pneumococcal carriage density and IL-17A levels.

Based on this difference in IL-17A levels between these two groups, we next examined whether there was a correlation between IL-17A levels and pneumococcal carriage density for all children. There was a significant inverse correlation between pneumococcal carriage density and IL-17A levels (N = 65; r = -0.32, p = 0.008; Fig 2A). However, there was a significant correlation between IL-17A levels in children with low pneumococcal carriage density (r = 0.40, p = 0.04; Fig 2B) but not in children with high pneumococcal carriage density (r = -0.07, p = 0.714; Fig 2C).

### Cytokine profile in children with low and high pneumococcal carriage densities

**Fig 3:**
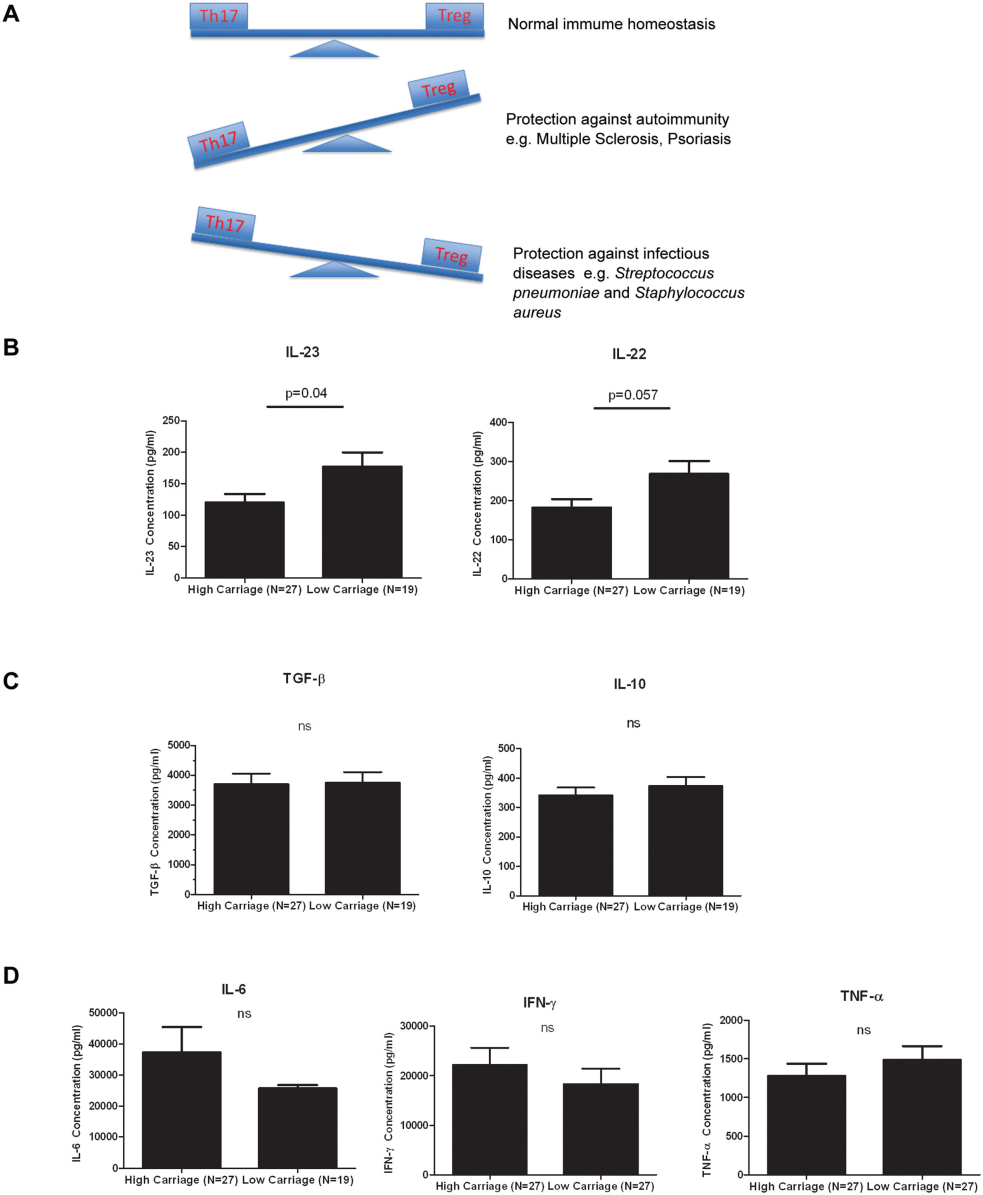
Cytokine profiles in PBMCs from children with low and high pneumococcal carriage densities. (A) Relationship between Th17 and Tregs under normal immune homeostasis and under different diseases contexts (Figure taken from [36]). Th17 cells are the major source of IL-17A which plays a protective role against pneumococcal infection, fungal infection and extracellular bacteria such as *Staphylococcus aureus*. However, in autoimmune and chronic inflammatory diseases such as Multiple sclerosis, rheumatoid arthritis, inflammatory bowel disease and psoriasis, IL-17A is known to be pathogenic (Adapted from [12,37,38]). Comparison of (B) IL-23 and IL-22 (Th17) (C) TGF-β and IL-10 (Tregs) (D) IL-6, IFN-γ and TNF-α (pro-inflammatory cytokines) levels between children with high or low pneumococcal carriage densities. Bars represent mean ± SEM. Statistical comparisons were done using an unpaired Student’s t test.

We examined the Th17 cytokine profile from children in Fiji with high (N = 27) and low (N = 27) pneumococcal carriage. There was a significant difference for IL-23 (1.5-fold, p = 0.04) while IL-22 was borderline significant (1.5-fold, p = 0.057) between the two carriage groups (Fig 3B). IL-21 was not detected (data not shown). No difference in the levels of other pro-inflammatory or Treg cytokines such as IFN-γ, IL-6, TNF-α, IL-10 and TGF-β between the two carriage groups were found (Fig 3C and 3D). Measurement of plasma cytokine levels did not reveal any detectable response (data not shown).

**Fig 4:**
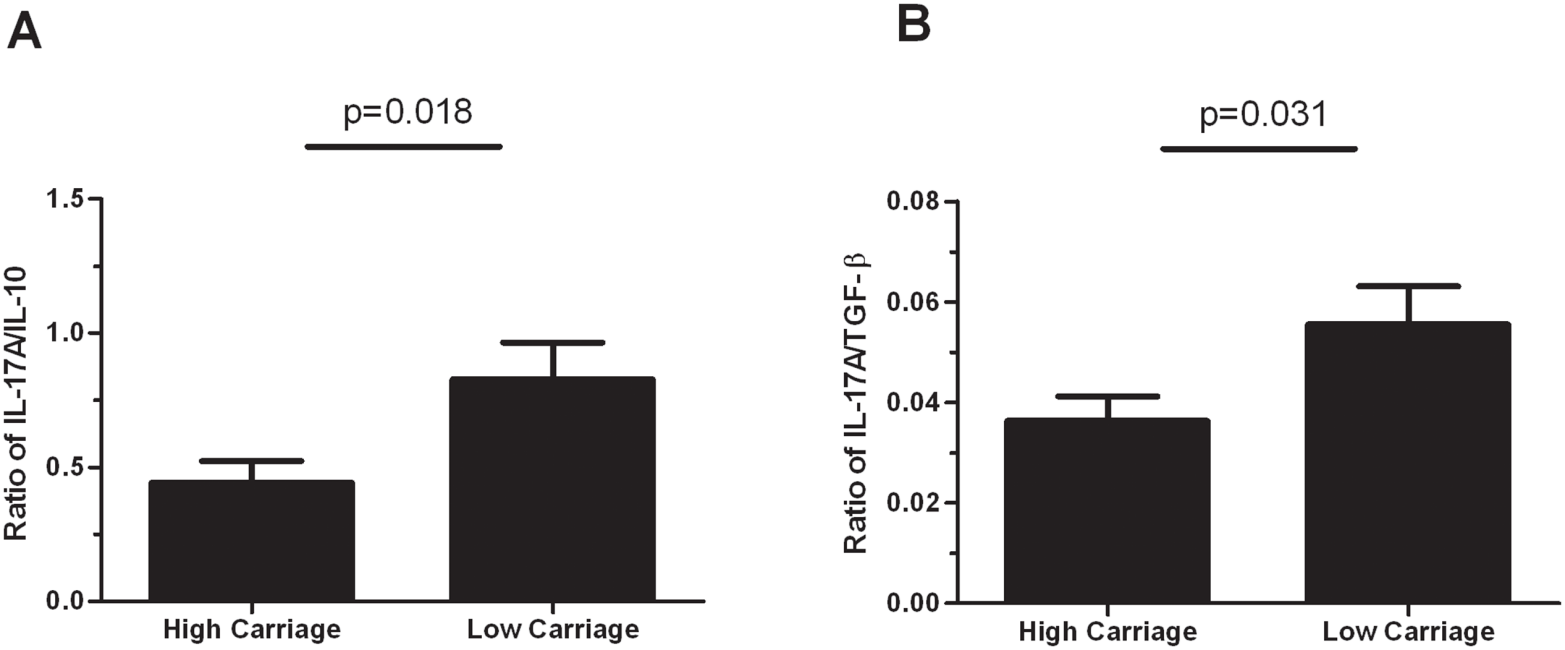
Th17/Treg balance is different in children with low and high pneumococcal carriage densities. The Th17/Treg axis is shown for IL-17A/IL-10 (A) and IL-17A/TGF-β (B) Bars represent mean ± SEM. Comparisons were done using an unpaired Student’s test.

Consistent with this, significantly higher Th17/Treg cytokine ratios in children with low pneumococcal carriage compared to children with high pneumococcal carriage were observed, with a fold change of 1.9 for IL-17/IL-10 (p = 0.018) and 1.5 for IL-17/TGF-β (p = 0.031) (Fig 4).

**Fig 5:**
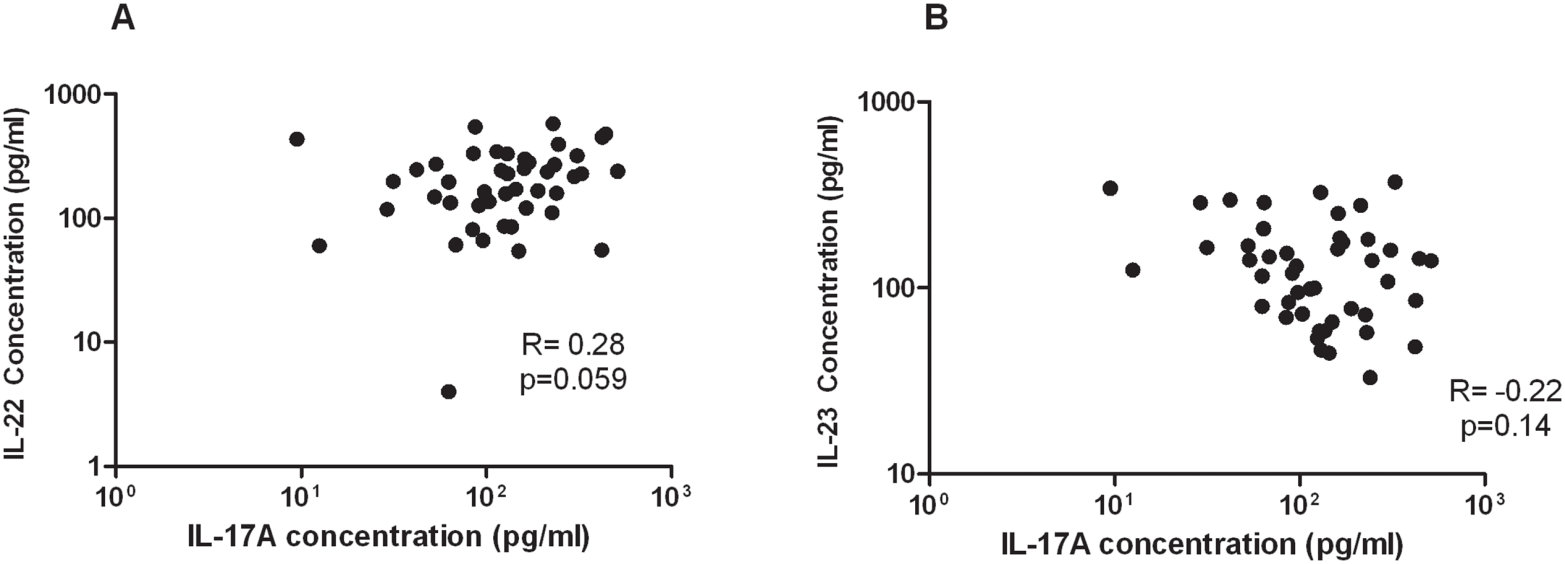
Correlation between IL-17A and other Th17 cytokines IL-22 (A) and IL-23 (B) levels in PBMCs from children with high and low pneumococcal carriage density (N = 46). The Spearman test was used to correlate the cytokine levels.

There was a close to significant positive correlation between IL-17A and IL-22 (r = 0.28, p = 0.059) (Fig 5A), while no correlation was observed between IL-17A and IL-23 levels (Fig 5B).

## Discussion

To our knowledge, this study is the first to characterise the relationship between Th17 responses and pneumococcal carriage density in children aged 5 to 7 years. We demonstrated that low pneumococcal carriage density was associated with increased IL-17A secretion in children. Prior studies have been in mouse models of pneumococcal colonisation and demonstrated a protective role for IL-17A against pneumococcal carriage [7,8,19]. Our findings add support to the emerging role of IL-17A in protecting against pneumococcal colonisation. As pneumococcal colonisation is a pre-requisite for the development of IPD, strategies that effectively target Th17 responses may be of significant benefit in reducing pneumococcal carriage in children at greatest risk of pneumococcal disease.

A number of studies have shown that CD4+ Th17 cells protect against pneumococcal carriage [9,19,20]. This role for IL-17A was reinforced by our data demonstrating higher IL-17A and other Th17-related cytokines (IL-22 and IL-23) in children with low pneumococcal carriage density compared to those with high carriage density. IL-17A is a signature cytokine produced by CD4+ Th17 cells and IL-21 and IL-22 are other cytokines produced by this subset [21,22]. In the context of pneumococcal diseases, IL-22 can induce production of anti-microbial peptides such as beta defensin 2 which can inhibit the growth of the pneumococcus, and is also important for protection against different infections including pneumonia caused by Gram-negative bacteria [23,24]. IL-23, although strictly not part of the Th17 pathway, promotes the development, proliferation and maintenance of Th17 cells [25]. Furthermore, Th17 and Treg cells are known to be reciprocally regulated during inflammation. Our data showed no difference in the Treg cytokines, IL-10 and TGF-β, between children with high or low pneumococcal carriage density. In addition, the higher Th17/Treg ratios associated with low density pneumococcal carriage further suggests an overall skewing towards the Th17 phenotype in these children.

Interestingly, we found a strong correlation between IL-17A and low pneumococcal carriage density but not with high pneumococcal carriage density. It is possible under conditions of low pneumococcal carriage density, IL-17A may be important in controlling the inflammatory process thereby limiting the spread of pneumococcus in the nasopharynx. In contrast, IL-17A responses may be less able to control pneumococcal carriage under high density conditions. Alternatively, dysregulated or ineffective IL-17A responses may provide a conducive environment to allow the pneumococcus to survive in greater numbers. Paradoxically, IL-17A levels were lower in children that did not carry pneumococcus compared with children with low pneumococcal carriage density. While it may be anticipated that these children would have higher IL-17A levels, this could be explained by the fact that IL-17A levels in these children may be due to inflammatory responses not related to the pneumococcus. Children at this age are known to carry other organisms in the nasopharynx such as *Haemophilus influenzae*, *Staphylococcus aureus* as well as a number of viruses [17,26,27]. Additionally, other factors such as exposure to the organism may be more important in determining whether colonisation occurs, whereas IL-17A may be involved in limiting colonisation density.

While the carriage thresholds used in this study demonstrated a difference in IL-17A levels, there is a lack of consensus on the definition of high or low carriage density. High pneumococcal load has been associated with disease severity, with one study showing a pneumococcal load above 10^5^ CFU/ml in serum was associated with increased mortality [28]. Another study showed that higher nasopharyngeal pneumococcal load was detected in Vietnamese children with radiologically confirmed pneumonia compared with healthy controls and children with lower respiratory tract infections [29]. Similarly, other studies have reported a correlation between nasopharyngeal pneumococcal load and disease severity disease, although IL-17A levels were either not measured or associated with respiratory disease outcomes [30,31,32]. The thresholds defined in this study were based on correlating pneumococcal carriage with IL-17A levels and supports a role for IL-17A in low pneumococcal carriage density. However, further studies in larger cohorts are needed to confirm this relationship. Determination of optimal carriage thresholds is dependent on a number of factors such as bacterial species, type of specimen collected, patient cohort, the disease setting and socio-economic status. Using a threshold of 1x10^6^ CFU/ml did not reveal any differences in IL-17A levels (data not shown), and found that adjusting this threshold and excluding some participants with mid-level colonisation was more useful to examine the role of IL-17A in pneumococcal colonisation. In addition, there was a significant difference in IL-17A levels when we examined the thresholds >10^6^ CFU/ml, N = 25 (high pneumococcal carriage density) and <10^5^ CFU/ml, N = 19 (low pneumococcal carriage density) (data not shown). While this gives us confidence, further studies in larger cohorts are needed to confirm the validity of these thresholds.

In Fiji, ethnicity has an impact on carriage of pneumococci and other bacterial species. The population of Fiji consists of 57% indigenous Fijians (known as i-Taukei) and 38% Indo-Fijians (of Indian descent), with iTaukei Fijians having higher rates of pneumococcal carriage [14,17]. In our study, the two carriage groups contained children that were predominantly iTaukei (85% and 96% for low and high carriage groups, respectively), suggesting that the differences in carriage densities were not attributed to ethnicity alone. As expected, the no carriage group had a higher proportion of Indo-Fijians and a lower proportion of iTaukei Fijians compared to other carriage groups. Furthermore, the differences in carriage density were not related to PCV7 or pneumococcal polysaccharide vaccine use as children in both groups had a similar vaccination history. Based on this, it is likely other biological factors between children in these groups such as immune function, host pathogen interactions, and exposure to pneumococcus may have contributed to the differences in carriage density observed. Here, we report that differences in IL-17A levels relate to colonization density, although other factors may be involved. It would be interesting to examine the relationship between pneumococcal serotypes, colonization density, and IL-17A, but a large sample size would be required for serotype-specific analyses. Likewise, a longitudinal study design would be needed to investigate the relationship of colonization and IL-17A over time.

Lundgren and co-workers were the first to characterise and compare Th17 responses in children and adults from a developed country (Sweden) and developing country (Bangladesh) [5]. However, pneumococcal carriage densities were not examined and so data could only be compared betweens populations with high and low pneumococcal exposure. These *ex vivo* human studies showed IL-17A is produced primarily by the CD4+ memory T cells, but not CD4+ naïve T cells, in response to pneumococcal whole cell antigen stimulation. The same study also found higher levels of IL-17A and a greater proportion of CD4+ memory T cells in children from Bangladesh than children from Sweden. The higher IL-17A levels in Bangladeshi children is possibly explained by a higher proportion of CD4+ memory T cells in this high risk population due to frequent infections during early life. Therefore, IL-17 secreting CD4+ memory T cells are important for protection and a potential marker or correlate of protection after exposure to pneumococci. In our study, we were unable to do these analyses due to lack of cells. IL-17 is produced by numerous cell populations including CD4+ Th17 cells, NK cells, γδ T cells, CD8+ T cells and innate lymphoid cells [33]. However, a recent study demonstrated B cells also secrete IL-17 during infection with the protozoan parasite *Trypanosoma cruzi* and confer protection against this parasitic infection [34]. In our study, the source of IL-17 was PBMCs, predominately a lymphocyte population.

The role of Th17 in mediating immunity against pneumococcal colonisation and disease is of significant interest, has led to the development of a killed whole cell pneumococcal vaccine (WCV). A recent study found that WCV conferred protection in mouse models of carriage by reducing colonisation in a CD4+ T cell, IL-17A mediated mechanism [8]. The WCV could be an alternative approach to current PCVs as this vaccine is based on an unencapsulated strain of pneumococcus containing multiple proteins such as PspA conserved across the majority of serotypes, and may potentially prevent or reduce serotype replacement [14,35]. Ongoing studies in populations at high risk of disease such as Indonesia and Kenya to evaluate the WCV immunogenicity and impact on carriage are a priority.

Several limitations of our study need to be addressed. Firstly, this study was conducted in a relatively small number of children and our results need to be confirmed in larger studies. Secondly, due to the small sample size, differences in cytokine responses between ethnicities (i-Taukei versus Indo-Fijians) could not be determined. As the majority of the children in this cohort are i-Taukei, the results will need to be confirmed in other ethnic groups or settings. Lastly, our Th17 cytokine responses were measured in PBMC supernatants rather than the mucosal compartment, which would better reflect the local inflammatory response in the nasopharynx and may correlate better with carriage density data. However, direct measurement of Th17 responses in the nasopharynx is more challenging, particularly in human studies. Overall, there is a need for future clinical studies that examine the role of Th17 immunity in pneumococcal carriage and disease.

## Conclusion

Our results add to the emerging role for Th17 mediated protection against pneumococcal colonisation: Higher IL-17A levels were associated with lower carriage density. Therefore, CD4+ Th17, IL-17 secreting B cells and Th17 family of cytokines may be important biomarkers in predicting long term protection against pneumococcal carriage. Our data in humans also supports the potential benefits of vaccine strategies that stimulate IL-17A-based protection such as WCV.

## Supporting Information

S1 Table Demographics of children from Fiji who were separated into high (>8.21 x 10^5^ CFU/ml) and low (<1.67 x 10^5^ CFU/ml) pneumococcal carriage groups. (DOCX)
