# Supplementary material for: Reduced IL-17A Secretion Is Associated with High Levels of Pneumococcal Nasopharyngeal Carriage in Fijian Children
Source: PLoS One. 2015 Jun 12;10(6):e0129199. doi: 10.1371/journal.pone.0129199 (PMC4466549; doi:10.1371/journal.pone.0129199)
Supplement: S1 Table — (DOCX) [file pone.0129199.s001.docx]

**S1 Table: Demographics of children from Fiji who were separated into high (>8.21 x 10^5^ CFU/ml**) **and low (<1.67 x 10^5^ CFU/ml) pneumococcal carriage groups.**

| **Characteristic** | **High Carriage (%)** | **Low Carriage (%)** | **No Carriage (%)** |
| --- | --- | --- | --- |
|  | **(N=27)** | **(N=27)** | **(N=29)** |
| Sex |  |  |  |
| Male | 8 (30%) | 14 (52%) | 13 (45%) |
| Female | 19 (70%) | 13 (48%) | 16 (55%) |
| Ethnicity |  |  |  |
| Indigenous Fijian | 23 (85%) | 26 (96%) | 20 (69%)* |
| Indo-Fijian | 3 (11%) | 1 (4%) | 8 (28%)* |
| Others | 1 (4%) | 0 (0%) | 1 (3%) |
| Number of prior PCV7 doses# |  |  |  |
| 1 | 9 (33%) | 6 (22%) | 4 (14%) |
| 2 | 13 (48%) | 13 (48%) | 18 (62%) |
| 3 | 5 (19%) | 8 (30%) | 7 (24%) |
| Received 23vPPV† |  |  |  |
| Yes | 13 (48%) | 13 (48%) | 14 (48%) |
| No | 14 (52%) | 14 (52%) | 15 (52%) |

# PCV7 given during infancy at 6, 10 14 weeks of age, and for some of these children includes a catch up dose at 24 months (for the 1 dose PCV7 group, there were 4, 8 and 5 children and for the 2 dose PCV7 group, there were 4, 6 and 8 children in the no, low and high carriage groups, respectively).

† 23vPPV = 23 valent pneumococcal polysaccharide vaccine given at 12 months of age

*P<0.01 compared to the low carriage group.
